# An Approach for Stabilizing Abnormal Neural Activity in ADHD Using Chaotic Resonance

**DOI:** 10.3389/fncom.2021.726641

**Published:** 2021-09-01

**Authors:** Sou Nobukawa, Nobuhiko Wagatsuma, Haruhiko Nishimura, Hirotaka Doho, Tetsuya Takahashi

**Affiliations:** ^1^Department of Computer Science, Chiba Institute of Technology, Chiba, Japan; ^2^Department of Information Science, Faculty of Science, Toho University, Chiba, Japan; ^3^Graduate School of Applied Informatics, University of Hyogo, Kobe, Japan; ^4^Faculty of Education, Teacher Training Division, Kochi University, Kochi, Japan; ^5^Research Center for Child Mental Development, Kanazawa University, Kanazawa, Japan; ^6^Department of Neuropsychiatry, University of Fukui, Fukui, Japan; ^7^Uozu Shinkei Sanatorium, Uozu, Japan

**Keywords:** attention-deficit hyperactivity disorder, neural network, feedback control, chaos-chaos intermittency, neurofeedback

## Abstract

Reduced integrity of neural pathways from frontal to sensory cortices has been suggested as a potential neurobiological basis of attention-deficit hyperactivity disorder. Neurofeedback has been widely applied to enhance reduced neural pathways in attention-deficit hyperactivity disorder by repeated training on a daily temporal scale. Clinical and model-based studies have demonstrated that fluctuations in neural activity underpin sustained attention deficits in attention-deficit hyperactivity disorder. These aberrant neural fluctuations may be caused by the chaos–chaos intermittency state in frontal-sensory neural systems. Therefore, shifting the neural state from an aberrant chaos–chaos intermittency state to a normal stable state with an optimal external sensory stimulus, termed chaotic resonance, may be applied in neurofeedback for attention-deficit hyperactivity disorder. In this study, we applied a neurofeedback method based on chaotic resonance induced by “reduced region of orbit” feedback signals in the Baghdadi model for attention-deficit hyperactivity disorder. We evaluated the stabilizing effect of reduced region of orbit feedback and its robustness against noise from errors in estimation of neural activity. The effect of chaotic resonance successfully shifted the abnormal chaos-chaos intermittency of neural activity to the intended stable activity. Additionally, evaluation of the influence of noise due to measurement errors revealed that the efficiency of chaotic resonance induced by reduced region of orbit feedback signals was maintained over a range of certain noise strengths. In conclusion, applying chaotic resonance induced by reduced region of orbit feedback signals to neurofeedback methods may provide a promising treatment option for attention-deficit hyperactivity disorder.

## 1. Introduction

Attention-deficit hyperactivity disorder (ADHD) is a behavioral disorder underscored by inattention, impulsivity, and hyperactivity. ADHD is one of the most common neurobehavioral disorders presenting for treatment in both children and adolescents (DuPaul et al., 1998; American Psychiatric Association, 2013). ADHD symptoms may cause serious psychological and social effects on patients' quality of life (Sonuga-Barke et al., 2013). During development in particular, impulsivity and hyperactivity become less apparent, whereas attention deficits persist in most patients (Achenbach et al., 1995; Hart et al., 1995; Mick et al., 2004; Mullane et al., 2011). Therefore, efficacious treatments to ameliorate attention deficits in ADHD are a critical unmet need.

Dysfunction in dopaminergic (Tripp and Wickens, 2008; Volkow et al., 2009; Wu et al., 2012) and noradrenergic neural systems (Rowe et al., 2005; Konrad et al., 2006; van Dongen-Boomsma et al., 2010) across extensive brain regions has been well-described as a biological basis of ADHD. In particular, deficits in attention function are associated with the reduced integrity of these neural pathways (Rowe et al., 2005; Konrad et al., 2006; van Dongen-Boomsma et al., 2010) (reviewed in Swanson et al., 2007; Mueller et al., 2017). To ameliorate attention deficits in ADHD, medications that block dopamine and norepinephrine reuptake such as methylphenidate and atomoxetine are widely used (Gibbins and Weiss, 2007; Wolraich et al., 2019) and have been demonstrated to significantly improve symptoms (Stevens et al., 2013). Nevertheless, their long-term effects have not been confirmed (Molina et al., 2009; Cunill et al., 2016).

Neurofeedback is a type of biofeedback involving self-regulation of brain function. Neurofeedback involves the detection and measurement of neural activity and the generation of a recurrent signal to enable enhancement of neural pathways (Bluschke et al., 2016; Bussalb et al., 2019; Rubia et al., 2019; Van Doren et al., 2019). Based on the theory of reduced integrity of neural pathways in ADHD (Rowe et al., 2005; Konrad et al., 2006; van Dongen-Boomsma et al., 2010) (reviewed in Swanson et al., 2007; Mueller et al., 2017), neurofeedback techniques have gained increasing interest as a non-pharmacological treatment (reviewed in Hammond, 2007; Sitaram et al., 2017; Hampson et al., 2019) and have been successfully applied to chronically enhance the reduced integrity of neural pathways in ADHD (Strehl et al., 2006; Gevensleben et al., 2010; Van Doren et al., 2019).

In addition to the theory of reduced neural pathway integrity in ADHD, both clinical and model-based studies have demonstrated that fluctuations in neural activity contribute to sustained attention deficits in ADHD (Baghdadi et al., 2015; Gonen-Yaacovi et al., 2016; Michelini et al., 2018). In clinical studies, large temporal fluctuations in neural activity were observed in ADHD patients in conditions both with and without sensory stimuli (Gonen-Yaacovi et al., 2016); these fluctuations reflect sustained attention deficits in ADHD (Michelini et al., 2018). A model-based study by Baghdadi et al. showed that the temporal fluctuate behaviors in neural activity corresponded to abnormal temporal profiles of attention levels in ADHD in a neural network model consisting of excitatory and inhibitory neural populations in frontal and sensory cortices (Baghdadi et al., 2015) (this model is termed the Baghdadi model in this study). Using their model, Baghdadi et al. further ascertained that these aberrant neural fluctuations arose from chaos-chaos intermittency (CCI) (reviewed in Anishchenko et al., 2007), in which an orbit with chaotic behaviors hops among separated attractor regions (Baghdadi et al., 2015). In particular, in the case that the feedback of neural pathway from the frontal cortex and sensory cortex becomes weak, this abnormal CCI neural activity easily appears (Baghdadi et al., 2015).

According to the non-linear feedback control theory, appropriate external feedback signals permit the transition of a system state with abnormal behaviors to a stable state, typified as chaos-controlling methods (reviewed in Schöll and Schuster, 2008; Nobukawa and Nishimura, 2020). Furthermore, it is well-established that neural activity underpinning attention-related functions can be activated by external sensory stimuli (Moore et al., 2003; Perrin et al., 2004; Vandewalle et al., 2006; Newman et al., 2016). Therefore, directly stabilizing abnormal CCI with external sensory stimuli based on non-linear feedback control may serve as another approach to already established neurofeedback methods that reinforce neural pathways in ADHD (Hammond, 2007; Sitaram et al., 2017; Hampson et al., 2019). To stabilize abnormal CCI, the synchronization of CCI against an external stimulus, termed chaotic resonance (Nishimura et al., 2000) (review in Anishchenko et al. (2007); Rajasekar and Sanjuán (2016); Nobukawa and Nishimura (2020)), is a plausible solution (Nobukawa et al., 2018, 2019b, 2020a; Nobukawa and Shibata, 2019; Doho et al., 2020). This is because synchronization against an external stimulus as the intended reference of neural activity may induce the transition of dysfunctional neural activity to healthy neural activity.

As a feedback control method to induce chaotic resonance by external signals, we previously proposed the “reduced region of orbit” (RRO) feedback method, which reduces the absolute local maximum and minimum values of non-linear map functions in dynamical systems to induce attractor-merging bifurcation where chaotic resonance emerges (Nobukawa et al., 2018). This method enables the control of chaotic resonance without the need to adjust internal neural parameters (Nobukawa et al., 2018). Therefore, by broadening the scope of application of chaotic resonance, this method opened novel avenues for utilizing chaotic resonance in neural systems (Nobukawa and Shibata, 2019; Nobukawa et al., 2019b; Doho et al., 2020) (reviewed in Nobukawa and Nishimura, 2020). In particular, the RRO feedback method achieves the transition of abnormal neural activity of bipolar disorder due to imbalance of excitatory and inhibitory neural populations (E/I imbalance) to healthy state (Doho et al., 2020).

In this context, we hypothesized that the chaotic resonance produced by the RRO feedback method would promote an efficacious neurofeedback method to improve dysfunctional neural activity in ADHD under pathological impairment of neural pathway from the sensory cortex to frontal cortex as well as E/I imbalance. To verify this hypothesis, we applied the RRO feedback method to induce chaotic resonance in the Baghdadi model for ADHD. We then evaluated the stabilizing effect of RRO feedback and its robustness against noise due to measurement errors.

## 2. Materials and Methods

### 2.1. Frontal and Sensory Neural System Composed of Excitatory and Inhibitory Neural Populations

The pathology of ADHD involves multiple complicated neural pathways associated with dopamine (Tripp and Wickens, 2008; Volkow et al., 2009; Wu et al., 2012) and noradrenaline neural systems, which project to widespread brain regions (Rowe et al., 2005; Konrad et al., 2006; van Dongen-Boomsma et al., 2010). In particular, abnormal frontal cortical activity has been reported to cause attention dysfunction (Murias et al., 2007; Cubillo et al., 2012). The abnormal frontal activity in ADHD patients is associated with reduced inhibitory neural activity and dopaminergic activity (Barkley, 1997; Nigg, 2001; Spronk et al., 2008; Volkow et al., 2009; Loskutova et al., 2010; Fisher et al., 2011). The Baghdadi model (Baghdadi et al., 2015) is a neural network model that reproduces the abnormal temporal behavior of attention levels, focusing on the pathological imbalance between excitatory (glutamatergic) and inhibitory (GABAergic) neural populations in the frontal cortex (Barkley, 1997; Nigg, 2001; Spronk et al., 2008; Volkow et al., 2009; Loskutova et al., 2010) and dysfunction of feedback loops from the sensory cortex to the frontal cortex (Mazaheri et al., 2010; Moriyama et al., 2012).

**Figure 1:**
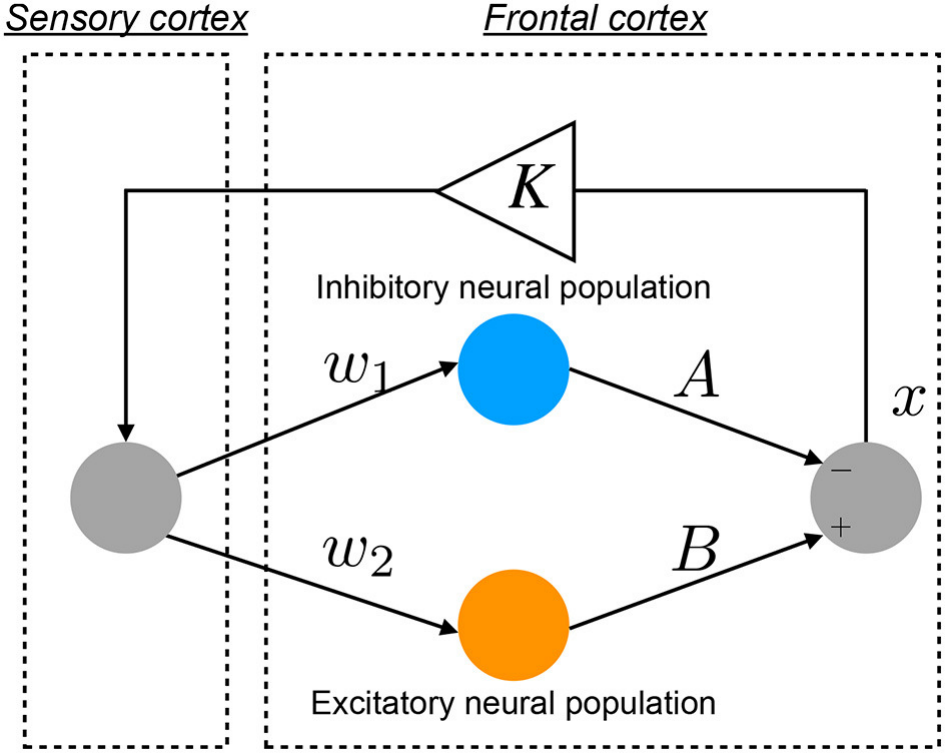
Neural network model to reproduce the abnormal temporal behavior of attention levels, focusing on the pathological imbalance between excitatory (glutamatergic) and inhibitory (GABAergic) neural populations in the frontal cortex and dysfunction in the feedback loop from the sensory cortex to frontal cortex (Baghdadi et al., 2015).

An overview of the Baghdadi model is presented in Figure 1. The temporal behavior of neural activity in the frontal cortex *x*(*n*) (*n* = 1, 2, ⋯) is regulated by the competition between excitatory and inhibitory neural populations (Baghdadi et al., 2015):

(1) $$\begin{array}{r} x\left(n+1\right)=F\left(x\left(n\right)\right), \end{array}$$

(2) $$\begin{array}{r} F\left(x\left(n\right)\right)=K\left(B\tanh\left(w_{2}x\left(n\right)\right)-A\tanh\left(w_{1}x\left(n\right)\right)\right). \end{array}$$

Here, *F*(*x*(*n*)) represents the map function for *x*(*n*). *w*_1_ and *A* indicate the synaptic weights of input and output for inhibitory neural populations, respectively. *w*_2_ and *B* represent the synaptic weights of input and output for excitatory neural populations, respectively. The positive and negative values of *x*(*n*) correspond to neural activities in the activate and resting state for neural population, respectively. *K* is an attenuation coefficient of frontal neural activity. In the Baghdadi model, frontal neural dynamics *x*(*n*) is determined by output from the frontal cortex: *B* tanh(*w*_2_*x*(*n*)−*A* tanh(*w*_1_*x*(*n*)) and its feedback through sensory cortex with attenuation *K* in Equation (2) (Baghdadi et al., 2015). Therefore, the output term from frontal cortex is multiplied by *K*. The setting of *K* < 1.0 corresponds to the case of the loss of information of brain activity due to lower attention (Baghdadi et al., 2015). In this study, we used the parameter set (*w*_1_ = 0.2223, *w*_2_ = 1.487) (Baghdadi et al., 2015).

### 2.2. Neural System With External Periodic Signals and RRO Feedback Signals

**Figure 2:**
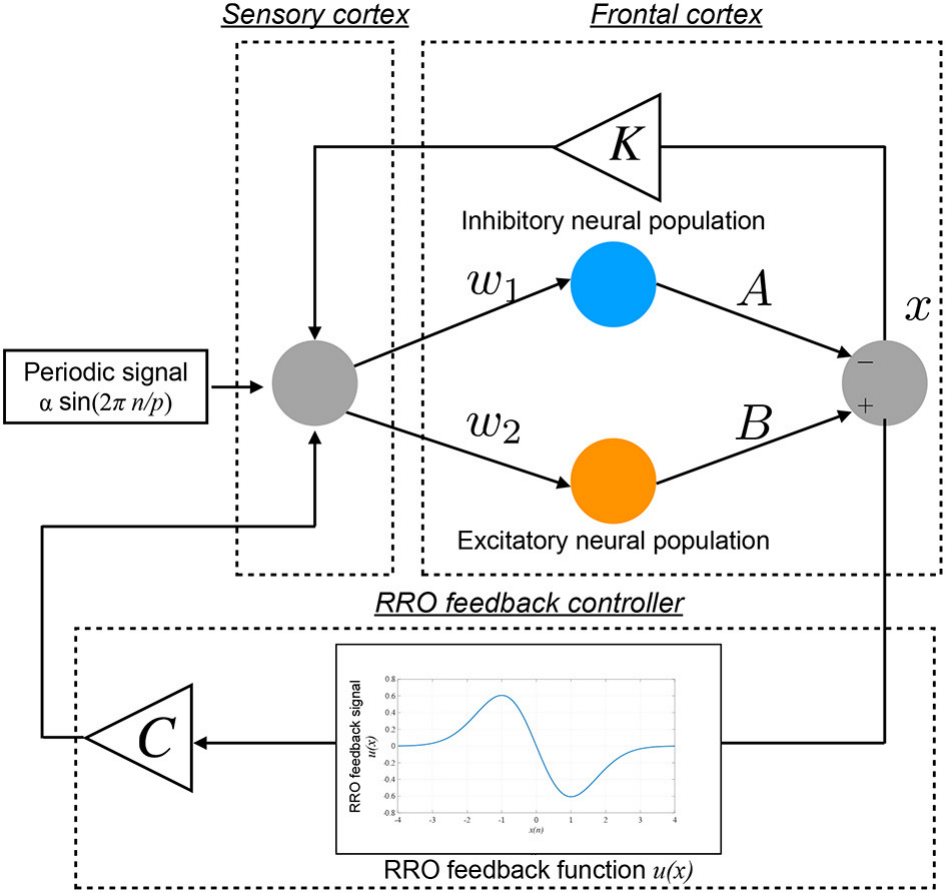
Neural system with the “reduced region of orbit” (RRO) feedback signals for inducing healthy neural activity. The frontal cortical neural activity *x*(*n*) is controlled by RRO feedback signals *Cu*(*x*) and a periodic input signal *S*(*n*) = α sin(2π*n*/*p*).

The conventional neurofeedback methods enhance the neural pathway by the repeated daily-temporal-scale training (Strehl et al., 2006; Gevensleben et al., 2010; Van Doren et al., 2019), which corresponds to increasing the strength of neural pathway *K* from the sensory cortex to the frontal cortex in the Baghdadi model. In this study, as another approach to directly stabilizing abnormal CCI, we applied the RRO feedback signals to the Baghdadi model to induce chaotic resonance for the transition of the CCI of *x*(*n*) to the periodic state. A methodological chart of the system for this control method is shown in Figure 2. The frontal cortical neural activity *x*(*n*) is controlled by RRO feedback signals *Cu*(*x*) and a periodic input signal *S*(*n*) = α sin(2π*n*/*p*), as follows:

(3) $$\begin{array}{r} x\left(n+1\right)=F\left(x\left(n\right)\right)+Cu\left(x\left(n\right)\right)+S\left(n\right), \end{array}$$

(4) $$\begin{array}{r} u\left(x\right)=-\left(x-x_{d}\right)\exp\left(-{\left(x-x_{d}\right)}^{2}/\left(2\sigma^{2}\right)\right). \end{array}$$

Here, *C*, *x*_*d*_, and σ denote the strength of RRO feedback, the merging point of two chaotic attractors, and a parameter to regulate the region of the RRO feedback effect, respectively. *S*(*n*) is an example reference of the desired neural activity corresponding to the healthy condition, i.e., the neural activity observed as lower temporal fluctuation in electroencephalogram (EEG) (Gonen-Yaacovi et al., 2016). We assumed that RRO feedback signal *Cu*(*x*(*n*)) and periodic input signal *S*(*n*) are implemented by an external sensory stimulus. In this study, we utilized *x*_*d*_ = 0 and σ = 1.0, because the structure of return-map of Equation (1) has a point symmetry at around *x* = 0 with local maximum and minimum values of the map function located within the region −σ < *x* < σ (σ = 1.0) (Nobukawa et al., 2018). For input signal *S*(*n*), we used the four *p* periods: 4, 8, 16, and 32.

To develop the RRO feedback signals based on actual frontal neural activity, the influence of measurement errors on RRO feedback signals must be evaluated. Therefore, in addition to the noise-free condition, we evaluated the influence of measurement errors on RRO feedback signals using Gaussian white noise ξ(*n*) (mean, 0; standard deviation, 1.0):

(5) $$\begin{array}{r} u_{e}\left(x\right)=-\left(\left(x+D\xi \left(n\right)\right)-x_{d}\right)\exp\left(-{\left(\left(x+D\xi \left(n\right)\right)-x_{d}\right)}^{2}/\left(2\sigma^{2}\right)\right). \end{array}$$

Here, *D* exhibits the noise strength.

### 2.3. Evaluation Indexes

To investigate neural activity, the bifurcation diagram of *x*(*n*) was used. To evaluate the chaotic state of the Baghdadi model, the Lyapunov exponent was measured by the following (Parker and Chua, 2012):

(6) $$\begin{array}{r} \lambda =\frac{1}{\tau M}\overset{M}{\underset{k=1}{\sum }}\ln\left(\frac{d^{k}\left(t_{l}=\tau \right)}{d^{k}\left(t_{l}=0\right)}\right). \end{array}$$

Here, $d^{k}\left(t_{l}=0\right)=d_{0}$ (*k* = 1, 2, ⋯ , *M*) indicates *M* perturbed initial conditions to the orbit of *x*(*n*) applied at *n* = *n*_0_ + (*k* − 1)τ. The temporal development during *t*_*l*_ ∈ [0 : τ] is $d^{k}\left(t_{l}=\tau \right)=\left(x\left(n\right)-x^{\prime }\left(n\right)\right){|}_{n=n_{0}+k\tau }$. *x*′(*n*) is an orbit-applied perturbation. The chaotic and periodic state of *x*(*n*) correspond to λ > 0 and λ < 0, respectively.

The CCI of *x*(*n*) is induced by attractor-merging bifurcation. To detect this bifurcation, the conditions *F*(*f*_max_) + *Cu*(*f*_max_) and *F*(*f*_min_) + *Cu*(*f*_min_) were utilized. *F*(*f*_max,min_) + *Cu*(*f*_max,min_) = 0 corresponds to the attractor-merging bifurcation point; in the attractor-merging condition, *F*(*f*_max_) + *Cu*(*f*_max_) < 0 and *F*(*f*_min_) + *Cu*(*f*_min_) > 0 are satisfied (Nobukawa et al., 2018).

To evaluate the synchronization between *x*(*n*) and *S*(*n*), we utilized their correlation coefficients with considering time delay τ:

(7) $$\begin{array}{r} \text{Corr}\left(\tau \right)=\frac{C_{sx}\left(\tau \right)}{\sqrt{C_{ss}C_{xx}}}, \end{array}$$

(8) $$\begin{array}{r} \;C_{sx}\left(\tau \right)=\langle \left(S\left(n+\tau \right)-\langle S\rangle \right)\left(X\left(n\right)-\langle X\rangle \right)\rangle , \end{array}$$

(9) $$\begin{array}{r} \;C_{ss}=\langle {\left(S\left(n\right)-\langle S\rangle \right)}^{2}\rangle , \end{array}$$

(10) $$\begin{array}{r} \;C_{xx}=\langle {\left(X\left(n\right)-\langle X\rangle \right)}^{2}\rangle , \end{array}$$

where 〈·〉 denotes the average in *n*. *X* represents the binarized *x*(*n*) value, i.e., *X*(*n*) = 1 in *x*(*n*) ≥ 0 case and *X*(*n*) = − 1 in *x*(*n*) < 0 to focus on the CCI behavior. In this study, τ was set to the value for arg max_τ_ Corr(τ) in each time series of *x*(*n*). arg max_τ_ Corr(τ) was assessed among ten trials using different initial conditions of *x*(0).

To evaluate the amount of perturbation for the applied signals consisting of input periodic signal *S*(*n*) and RRO feedback signal *Cu*(*x*), the following perturbation was used:

(11) $$\begin{array}{r} \Theta =<S{\left(n\right)}^{2}+{\left(Cu\left(x\left(n\right)\right)\right)}^{2}>, \end{array}$$

where < · > is the average in *n* (Doho et al., 2020). Θ was assessed among ten trials using different initial conditions of *x*(0).

## 3. Results

### 3.1. System Behavior in Neural Network Composed of Excitatory and Inhibitory Neural Populations

**Figure 3:**
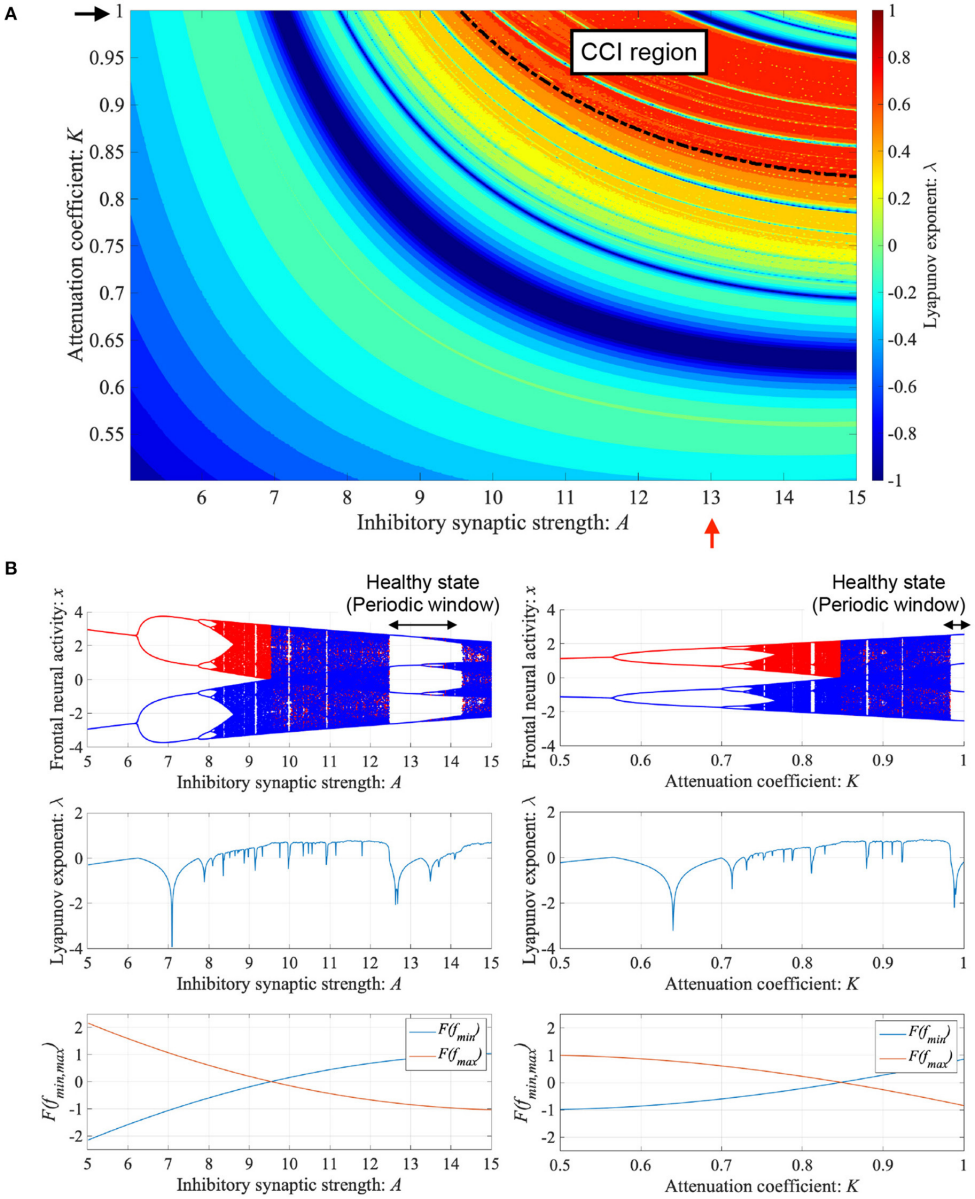
Dependence of system behavior on inhibitory synaptic strength *A* and attenuation coefficient of frontal neural activity *K* in the Baghdadi model. **(A)** Lyapunov exponent λ as a function of *A* and *K* (*B* = 5.821, *w*_1_ = 0.2223, *w*_2_ = 1.487). Black dashed line indicates attractor-merging condition *F*(*f*_max,min_) = 0. In λ > 0 in the upper region of the dashed black line satisfying the breaking attractor-merging condition, i.e., *F*(*f*_max_) < 0, *F*(*f*_min_) > 0, chaos-chaos intermittency (CCI), which corresponds to abnormal neural activity in ADHD, is noted. **(B)** (Left) Dependence of system behavior on *A* at *K* = 1.0 corresponding to *K* value indicated by black arrow in **(A)**. Bifurcation diagram of frontal neural activity *x* (top). Lyapunov exponent λ (middle). Attractor-merging condition *F*(*f*_max,min_) (bottom). (Right) Dependence of system behavior on *K* at *A* = 13.0 corresponding to *A* value indicated by red arrow in **(A)**. Bifurcation diagram of frontal neural activity *x* (top). The blue and red dots indicate the cases with positive and negative initial values *x*(0), respectively. Lyapunov exponent λ (middle). Attractor-merging condition *F*(*f*_max,min_) (bottom). Periodic windows in 12.2 ≲ *A* ≲ 14.3 (left panel of figure) and 0.87 ≲ *K* ≤ 1.0 (right panel of figure) correspond to the regions of healthy neural activity.

ADHD is characterized by an imbalance of the reduction in inhibitory neural activity caused by dysfunction in the dopaminergic neural system (Barkley, 1997; Nigg, 2001; Spronk et al., 2008; Volkow et al., 2009; Loskutova et al., 2010) and reduced feedback strength from the sensory cortex to frontal cortex (Moriyama et al., 2012). The neural activity of ADHD detected by EEG represents larger fluctuation in comparison with healthy condition (Gonen-Yaacovi et al., 2016). Baghdadi et al. demonstrated that this larger temporal fluctuation in ADHD and smaller temporal fluctuation in healthy condition might correspond to CCI and periodic behaviors in their proposed model (Baghdadi et al., 2015). First, we demonstrated the dependence of system behavior on inhibitory synaptic strength *A* and attenuation coefficient of frontal neural activity *K* in the Baghdadi model. Figure 3A shows the Lyapunov exponent λ as a function of *A* and *K* and attractor-merging condition *F*(*f*_max,min_) = 0 in the case of (*B* = 5.821, *w*_1_ = 0.2223, *w*_2_ = 1.487). In the region for breaking the attractor-merging condition, i.e., *F*(*f*_max_) < 0, *F*(*f*_min_) > 0 and arising chaotic activity λ>0, CCI, which corresponds to abnormal neural activity in ADHD, emerges. The system behavior under the conditions of fixed *K* or *A* values is depicted in Figure 3B. The left and right panels of Figure 3B show the dependence of system behavior on *A* at *K* = 1.0 and dependence on *K* at *A* = 13.0, respectively, by the bifurcation diagram of frontal neural activity *x*, Lyapunov exponent λ, and attractor-merging condition *F*(*f*_max,min_). The results demonstrate that CCI (*F*(*f*_max_) < 0, *F*(*f*_min_) > 0, λ>0) corresponding to abnormal neural activity in ADHD, which was demonstrated by Baghdadi et al. (2015), arises in the region 9.8 ≲ *A* ≲ 12.3 and *A* ≳ 14.3 in the dependence on *A* and 0.85≲ *K* ≲ 0.98 in the dependence on *K*. Furthermore, in the adjacent CCI regions, periodic windows in 12.2 ≲ *A* ≲ 14.3 and 0.87 ≲ *K* ≤ 1.0, corresponding to the regions for healthy neural activity (Baghdadi et al., 2015), exist.

### 3.2. Controlling Abnormal Neural Activity Using the RRO Feedback Method

**Figure 4:**
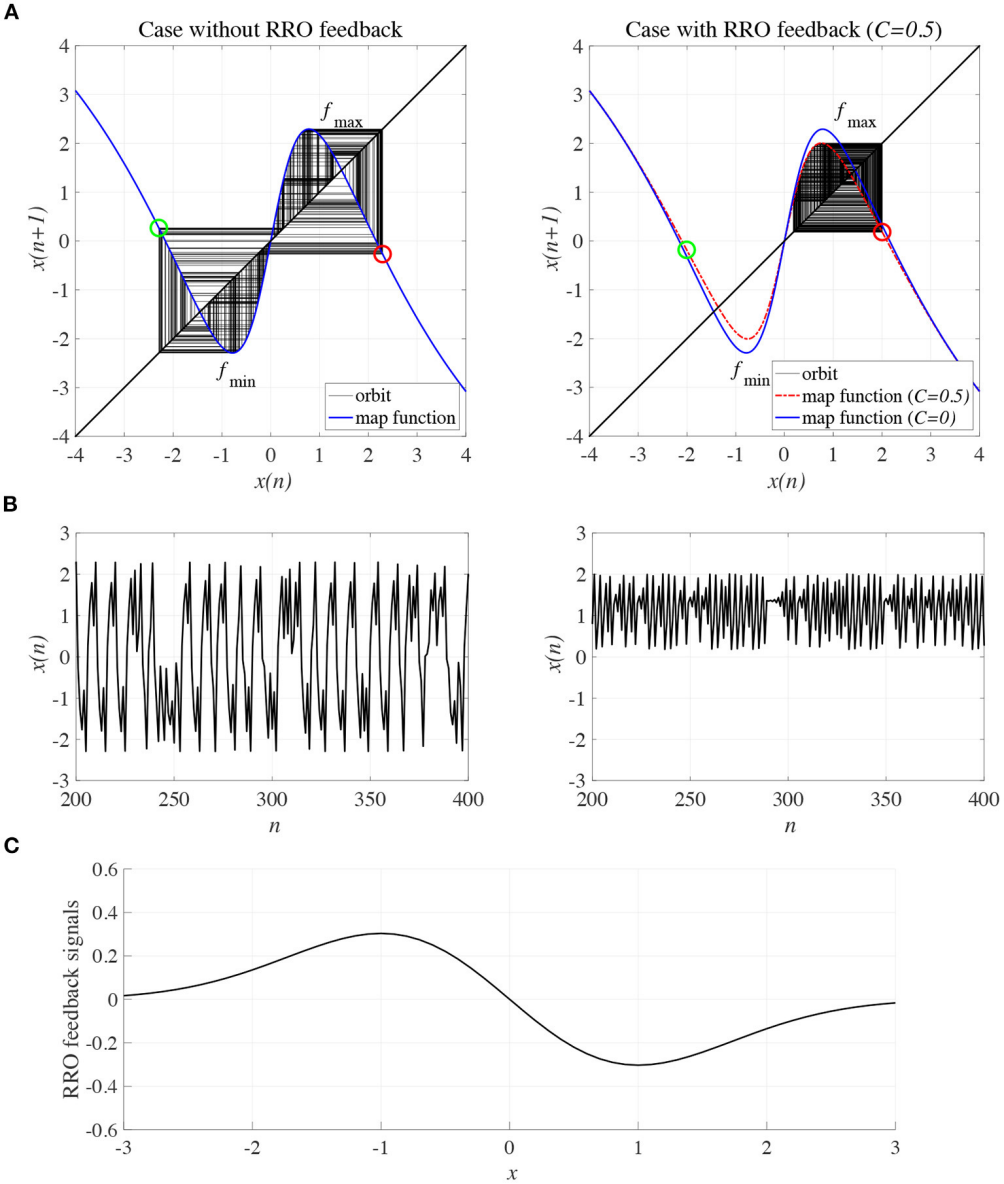
Controlling CCI by “reduced region of orbit” (RRO) feedback signals in the Baghdadi model (*A* = 13.0, *B* = 5.821, *w*_1_ = 0.2223, *w*_2_ = 1.487, *K* = 0.9). **(A)** Map function of the Baghdadi model with RRO feedback signals given by Equations (3) and (4) and its orbits in the case with feedback strength *C* = 0 (left panel) and 0.5 (right panel). Red and green open circles indicate attractor-merging conditions *F*(*f*_max,min_) + *Cu*(*f*_max,min_) (red: *f*_max_ case, green: *f*_min_ case). In *C* = 0 case, the attractor-merging conditions: *F*(*f*_max_) + *Cu*(*f*_max_) < 0 and *F*(*f*_min_) + *Cu*(*f*_min_) > 0 is satisfied. While, in *C* = 0.5 case, the attractor is separated due to *F*(*f*_max_) + *Cu*(*f*_max_) > 0 and *F*(*f*_min_) + *Cu*(*f*_min_) < 0. **(B)** Time series of *x*(*n*) corresponding to the orbits given by **(A)** in the case with feedback strength *C* = 0 (left panel) and 0.8 (right panel). **(C)** Profile of RRO feedback signals in the case with *C* = 0.5. CCI in the temporal behavior of *x*(*n*) is restricted, and the orbit is confined to either the negative or positive regions of *x*(*n*), depending on the initial value of *x*(0).

**Figure 5:**
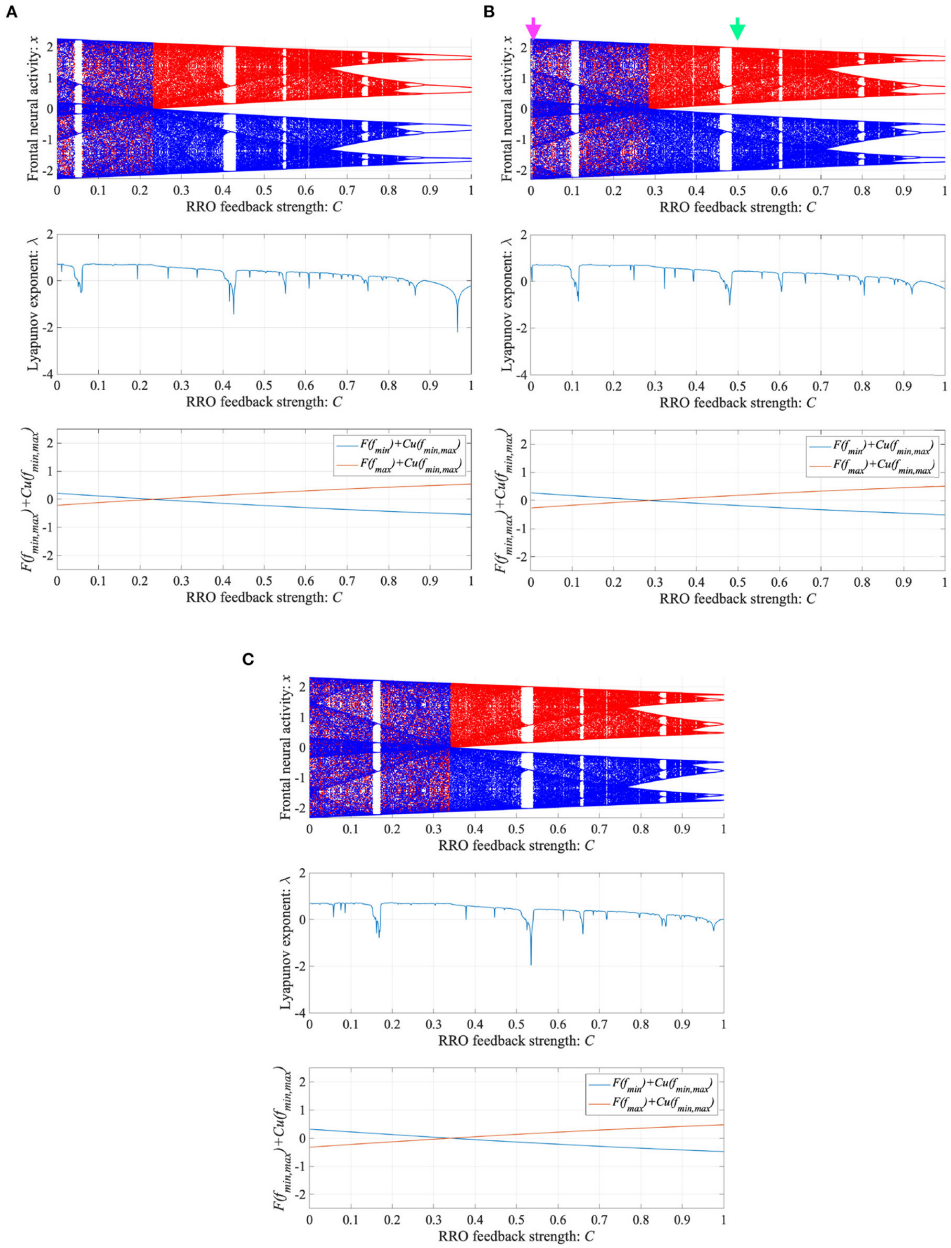
Dependence of system behavior on RRO feedback strength *C* in the Baghdadi model. (*A* = 13.0, *B* = 5.821, *w*_1_ = 0.2223, *w*_2_ = 1.487, *K* = 0.9). Bifurcation diagram of frontal neural activity *x* (top). Lyapunov exponent λ (middle). Attractor-merging condition *F*(*f*_max,min_) + *Cu*(*f*_max,min_) (bottom). **(A)** Attenuation *K* = 0.89. **(B)** *K* = 0.9. Here, magenta and green arrows correspond to the parameter sets for attractor merging (*C* = 0) and separated (*C* = 0.5) conditions in Figure 4. **(C)** *K* = 0.91. CCI behavior between positive and negative *x* regions (*F*(*f*_max_) + *Cu*(*f*_max_) < 0, *F*(*f*_min_) + *Cu*(*f*_min_) > 0, λ > 0) is suppressed (*F*(*f*_max_) + *Cu*(*f*_max_) > 0, *F*(*f*_min_) + *Cu*(*f*_min_) < 0) in the region of feedback strength *C* ≳ 0.23, 0.28, 0.34 in the cases with *K* = 0.89, 0.9, 0.91, respectively.

To control CCI behaviors caused by the abnormal imbalance in excitatory and inhibitory neural activity and the weaker feedback of neural pathways from the frontal and sensory cortices shown in Figure 3, RRO feedback signals were applied to the Baghdadi model according to Equations (3) and (4). Figure 4 shows the map function of the Baghdadi model with RRO feedback signals and its orbits (see Figure 4A), the time-series of *x*(*n*) (see Figure 4B) in the case with feedback strength *C* = 0, 0.5, and the profile of RRO feedback signals with its strength *C* = 0.5 (see Figure 4C). In the *C* = 0.5 case, the absolute local maximum/minimum values of map functions *f*_max,min_ were reduced by the effect of RRO feedback signals. By this reduction, the attractor-merging condition was broken, i.e., *F*(*f*_max_) + *Cu*(*f*_max_) > 0 and *F*(*f*_min_) + *Cu*(*f*_min_) < 0; consequently, *x*(*n*) stayed in either the positive or negative region of *x*(*n*). The dependence of the system behavior on RRO feedback strength *C* was evaluated in more detail. Figure 5 shows the bifurcation diagram of *x*, Lyapunov exponent λ, and attractor-merging condition *F*(*f*_max,min_) + *Cu*(*f*_max,min_) in attenuation *K* = 0.89, 0.9, 0.91. CCI behavior between positive and negative *x* regions (*F*(*f*_max_) + *Cu*(*f*_max_) < 0, *F*(*f*_min_) + *Cu*(*f*_min_) > 0, λ>0) was suppressed (*F*(*f*_max_) + *Cu*(*f*_max_) > 0, *F*(*f*_min_) + *Cu*(*f*_min_) < 0) in the region of feedback strength *C* ≳ 0.23, 0.28, 0.34 in the cases with *K* = 0.89, 0.9, 0.91, respectively. Additionally, the periodic windows appear at around *C* = 0.05, 0.1, 0.15, in the cases with *K* = 0.89, 0.9, 0.91. However, the RRO feedback signal does not always produce these periodic windows with lower temporal fluctuation corresponding to healthy condition. Therefore, the external periodic input *S*(*n*) is needed for the transition to the lower temporal fluctuation, which is dealt in section 3.3.

### 3.3. Transition of Abnormal Neural Activity to Healthy State by Synchronization

**Figure 6:**
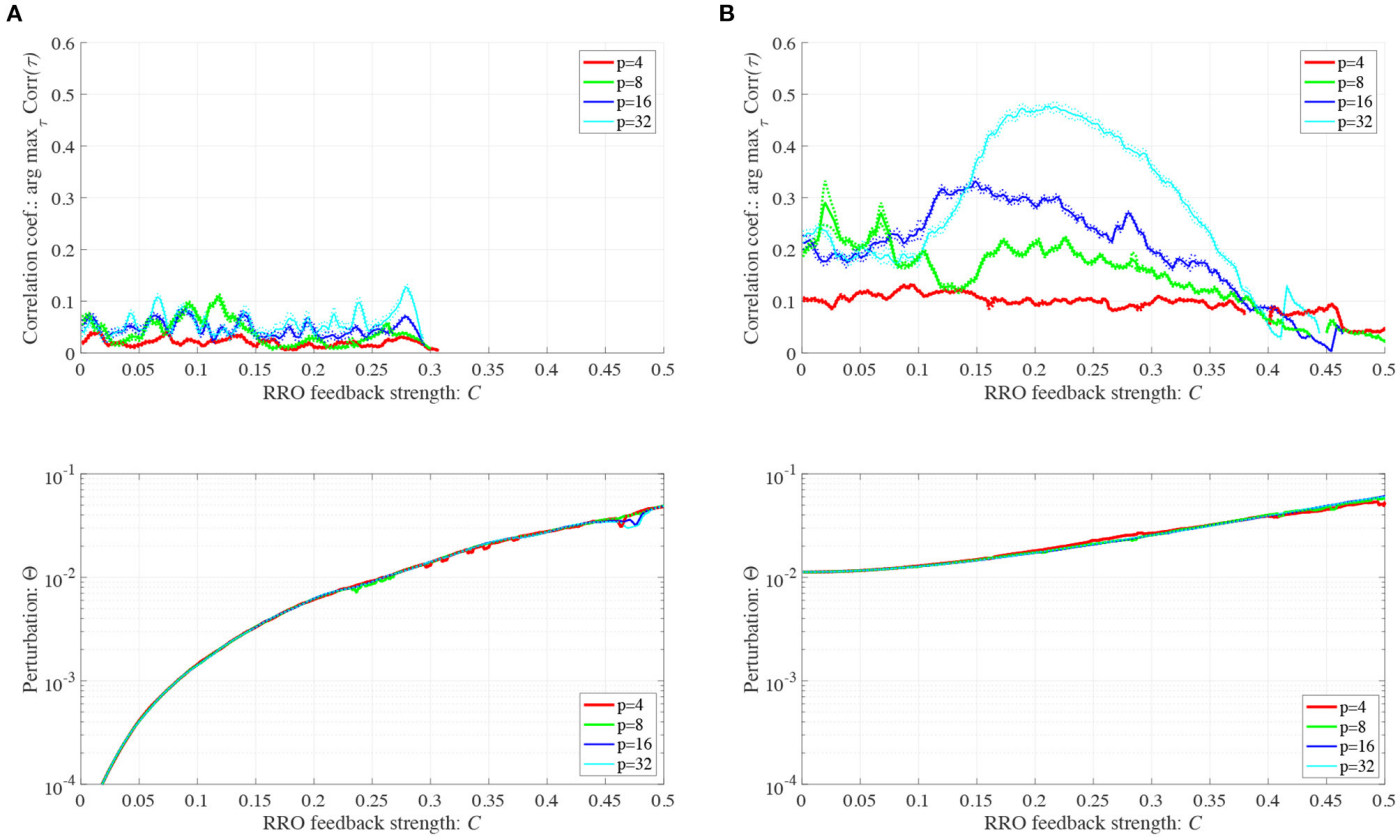
Synchronization against external periodic input induced by RRO feedback signals. Dependence of correlation coefficient arg max_τ_ Corr(τ) between *x*(*n*) and a periodic input signal *S*(*n*) = α sin(2π*n*/*p*) on the strength of RRO feedback signals *C* (top). Dependence of perturbation Θ on the strength of RRO feedback signals *C* (bottom). (*A* = 13.0, *B* = 5.821, *w*_1_ = 0.2223, *w*_2_ = 1.487, *K* = 0.9). **(A)** Case for the input signal strength α = 0.01. **(B)** Case for the input signal strength α = 0.15. Here, solid and dotted lines indicate mean and standard deviation, respectively. In α = 0.15 and relatively long periods such as *p* = 16, 32, the high synchronization (arg max_τ_ Corr(τ) ≈ 0.3, 0.5 in *p* = 16, 32, respectively) is produced by RRO feedback signals at its appropriate strength *C* = 0.2, where its perturbation Θ is significantly smaller (Θ ≈ 0.02) than the temporal variation in *x*(*n*) shown in Figure 5.

**Figure 7:**
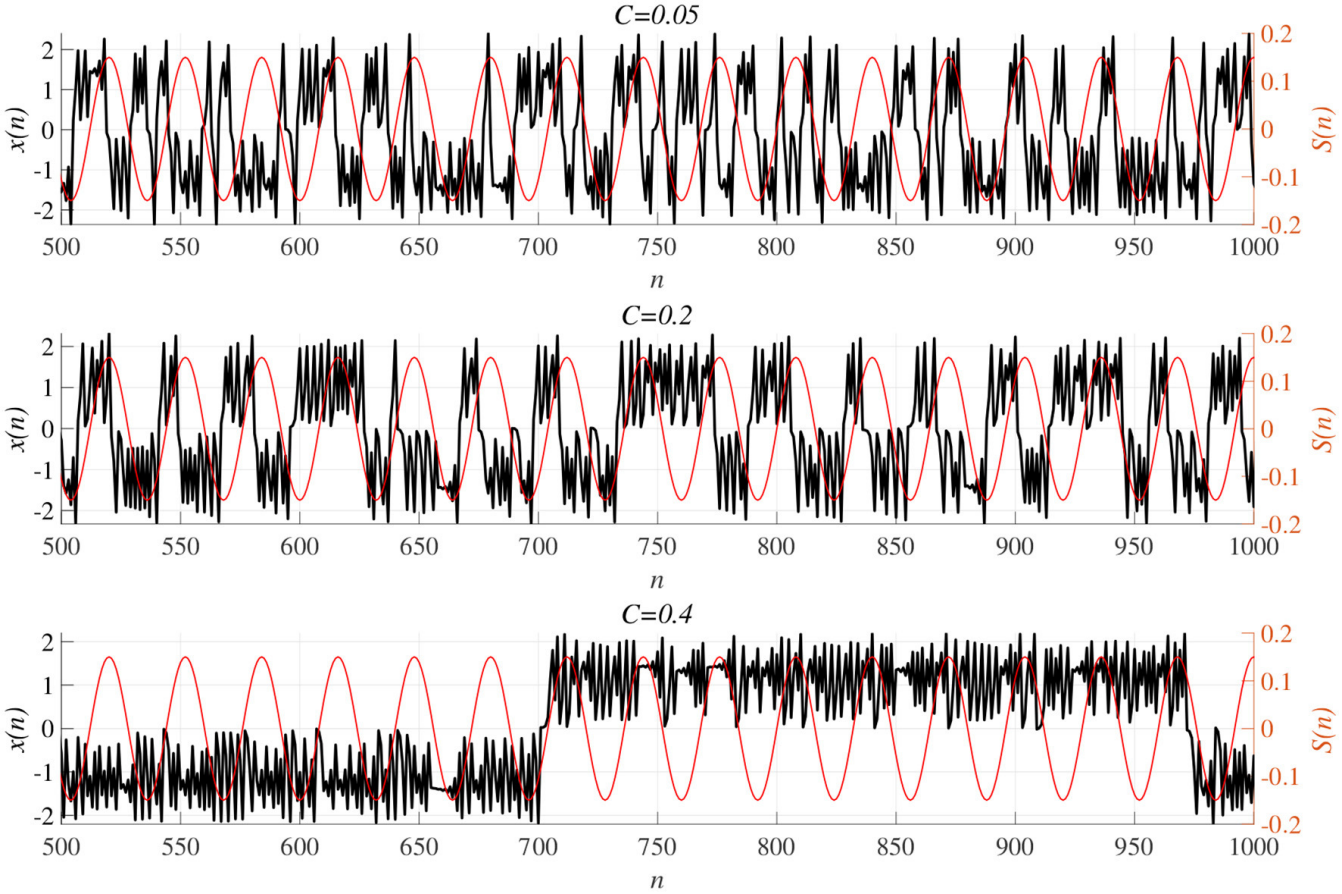
Time-series of *x*(*n*) (black line) under RRO feedback signals *Cu*(*x*) and periodic input signal *S*(*n*) (red line) corresponding to the case of α = 0.15, *p* = 32 in Figure 6. **(Top)** A case of strength of RRO feedback signals *C* = 0.05. (middle) *C* = 0.2 case. **(Bottom)** *C* = 0.4 case. Under small RRO feedback signals (*C* = 0.05), the frequency of CCI is too high; subsequently, CCI does not synchronize to *S*(*n*) (correlation coefficient arg max_τ_ Corr(τ) ≈ 0.23). In contrast, under the appropriate RRO feedback strength *C* = 0.2, the frequency of CCI is reduced. Using the appropriate CCI frequency enables high synchronization to be achieved (arg max_τ_ Corr(τ) ≈ 0.46). Under stronger RRO feedback strength, CCI does not respond to *S*(*n*) (arg max_τ_ Corr(τ) ≈ 0.06) due to CCI frequency being too low.

We investigated synchronization against external periodic input *S*(*n*) induced by RRO feedback signals. The top panel of Figure 6 shows the dependence of correlation coefficient arg max_τ_ Corr(τ) between *x*(*n*) and a periodic input signal *S*(*n*) on the strength of RRO feedback signals *C* (*A* = 13.0, *B* = 5.821, *w*_1_ = 0.2223, *w*_2_ = 1.487, *K* = 0.9) in cases of input signal strength α = 0.01, 0.15. In α = 0.15 and relatively long periods, such as *p* = 16, 32, the high synchronization (arg max_τ_ Corr(τ) ≈ 0.3, 0.5 in *p* = 16, 32, respectively) was produced by RRO feedback signals at its appropriate strength *C* = 0.2. This strength *C* = 0.2 corresponded to one for slightly weaker strength of attractor-merging bifurcation *F*(*f*_max,min_) + *Cu*(*f*_max,min_) = 0 at *C* = 0.28 under condition without the external periodic stimulus *S*(*n*) (see Figure 5). Here, by the additional effect of *S*(*n*), the attractor-merging bifurcation appears at its appropriate strength *C* = 0.2. That is, chaotic resonance was interpreted as being induced by RRO feedback signals and external stimulus *S*(*n*) (Nobukawa et al., 2018, 2019b; Nobukawa and Shibata, 2019). The dependence of perturbation Θ of *S*(*n*) and *Cu*(*x*(*n*)) on the strength of RRO feedback signals *C* is shown in the bottom panels of Figure 6. The results indicated that Θ to achieve the high synchronization state was significantly smaller (Θ ≈ 0.02) than the temporal variation in *x*(*n*) shown in Figure 5. As the typical time-series of *x*(*n*), Figure 7 shows the time-series of *x*(*n*) under RRO feedback signals *Ku*(*x*) and periodic input signal *S*(*n*) corresponding to the case of α = 0.15, *p* = 32 in Figure 6. Under small RRO feedback signals (*C* = 0.05), the frequency of CCI was too high; subsequently, CCI did not synchronize to *S*(*n*) (correlation coefficient arg max_τ_ Corr(τ) ≈ 0.23). In contrast, under the appropriate RRO feedback strength *C* = 0.2, the frequency of CCI was reduced. Using the appropriate CCI frequency, high synchronization was achieved (arg max_τ_ Corr(τ) ≈ 0.46). Under stronger RRO feedback strength (*C* = 0.4), CCI did not respond to *S*(*n*) (arg max_τ_ Corr(τ) ≈ 0.06) due to the CCI frequency being too low. In addition, at *C* = 0.4 under condition without *S*(*n*), the CCI does not appear (see *K* = 0.9 case in Figure 5), while the effect of the external stimulus *S*(*n*) leads CCI, although its frequency is low in *C* = 0.4 case of Figure 7.

**Figure 8:**
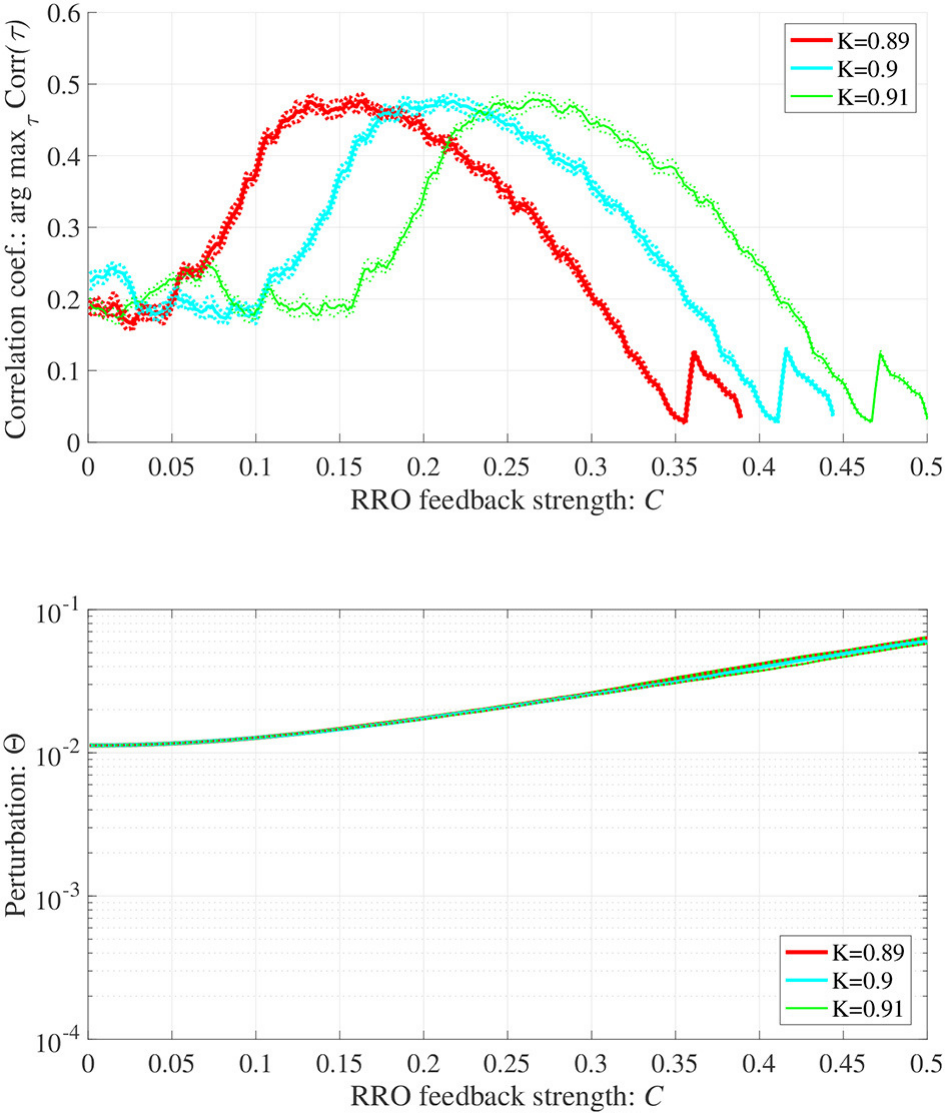
Synchronization against external periodic input induced by RRO feedback signals in cases with attenuation *K* = 0.89, 0.9, 0.91. Dependence of correlation coefficient arg max_τ_ Corr(τ) between *x*(*n*) and a periodic input signal *S*(*n*) = α sin(2π*n*/*p*) on the strength of RRO feedback signals *C* **(top)**. Dependence of perturbation Θ on the strength of RRO feedback signals *C* **(bottom)** (*A* = 13.0, *B* = 5.821, *w*_1_ = 0.2223, *w*_2_ = 1.487, *alpha* = 0.15, *p* = 32). Here, the solid and dotted lines indicate mean and standard deviation, respectively. With increasing *K*, attractor merging bifurcation point shifts to smaller *C* values (see Figure 5); subsequently, the peak of arg max_τ_ Corr(τ) shifts to smaller *C* region. At the different attenuation levels, the perturbation Θ to induce peak of arg max_τ_ Corr(τ) is significantly smaller (Θ ≈ 0.02) than the temporal variation in *x*(*n*) shown in Figure 5.

In addition to attenuation *K* = 0.9, the dependences of arg max_τ_ Corr(τ) and Θ at different levels of attenuation *K* = 0.89, 0.91 were evaluated under the same setting for *S*(*n*) (*p* = 32, α = 0.15 corresponding to Figure 6B) as shown in Figure 8. As the result, with increasing *K*, attractor merging bifurcation point shifts to smaller *C* values (see Figure 5); subsequently, the peak of arg max_τ_ Corr(τ) shifts to smaller *C* region. At these attenuation levels, the perturbation Θ to induce peak of arg max_τ_ Corr(τ) is significantly smaller (Θ ≈ 0.02) than the temporal variation in *x*(*n*) shown in Figure 5.

**Figure 9:**
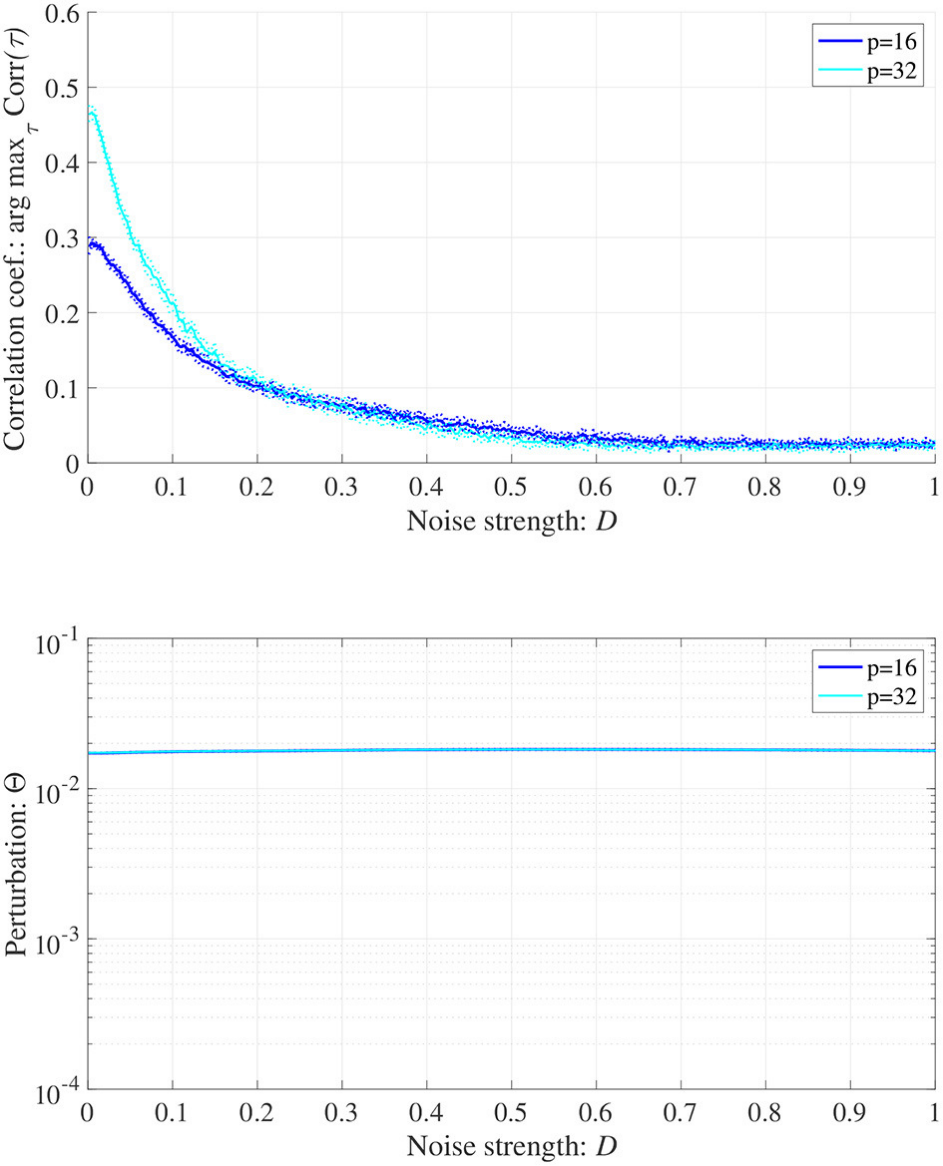
Synchronization against external periodic input induced by RRO feedback signals under Gaussian white noise *Dξ*(*n*). Here, RRO feedback strength *C* is fixed *C* = 0.2, where arg max_τ_ Corr(τ) exhibits a peak in Figure 6. Dependence of correlation coefficient arg max_τ_ Corr(τ) between *x*(*n*) and a periodic input signal *S*(*n*) = α sin(2π*n*/*p*) on noise strength *D* **(top)**. Dependence of perturbation Θ on noise strength *D* **(bottom)**. Here, the solid and dotted lines indicate mean and standard deviation, respectively. arg max_τ_ Corr(τ) decreases with increasing noise strength *D*, maintaining Θ ≈ 0.02. (*A* = 13.0, *B* = 5.821, *w*_1_ = 0.2223, *w*_2_ = 1.487, *K* = 0.9, α = 0.15).

When determining the RRO feedback signals estimated from actual frontal neural activity, measurement errors may affect the accuracy of producing RRO feedback signals. Therefore, we evaluated synchronization against external periodic input induced by RRO feedback signals under Gaussian white noise *Dξ*(*n*) given by Equation (5). Here, RRO feedback strength *C* is fixed *C* = 0.2 where arg max_τ_ Corr(τ) exhibits a peak in Figure 6B. Figure 9 shows the dependences of correlation coefficient arg max_τ_ Corr(τ) and perturbation Θ on noise strength *D* (*A* = 13.0, *B* = 5.821, *w*_1_ = 0.2223, *w*_2_ = 1.487, *K* = 0.9, α = 0.15). The results indicated that arg max_τ_ Corr(τ) decreased with increasing noise strength *D*, maintaining Θ ≈ 0.02.

## 4. Discussion and Conclusions

In this study, we developed an efficacious neurofeedback method based on chaotic resonance produced by RRO feedback signals in the Baghdadi model for abnormal neural activity in ADHD with E/I imbalance and impairment of neural pathway from the sensory cortex to the frontal cortex. We confirmed that the effect of chaotic resonance shifted aberrant neural activity caused by abnormal CCI of neural activity to healthy neural activity when the frequency of reference signals was relatively low. Moreover, we evaluated the influence of noise due to measurement errors and observed that the efficiency of chaotic resonance produced by RRO feedback signals was maintained over a certain range of noise strengths.

First, we discuss why the synchronization of CCI against the external reference signal is enhanced at attractor-merging bifurcation induced by RRO feedback signals, i.e., why chaotic resonance arises. Near the attractor-merging bifurcation, the frequency of autonomous CCI is low. In this condition, the external signal plays a perturbative role and switches neural activity between positive and negative attractor regions even if its strength is weak. Therefore, the CCI induced by the external signal becomes dominant among all CCIs; subsequently, high CCI synchronization with the external signal is realized. This tendency is congruent with our previous findings on chaotic resonance induced by RRO feedback signals (Nobukawa et al., 2018; Nobukawa and Shibata, 2019; Doho et al., 2020).

Then, we compare the current approach with conventional neurofeedback methods. The attractor-merging bifurcation induced by changing the synaptic weights as internal neural system parameters (see Figure 3) may correspond to the enhancement of neural pathways induced by the repeated daily-temporal-scale training used in conventional neurofeedback (Hammond, 2007; Baghdadi et al., 2015; Sitaram et al., 2017; Hampson et al., 2019). This is because abnormal CCI of neural activity is significantly suppressed under the condition of enhanced synaptic weights of the neural pathway from the frontal and sensory cortices, as reported in the model-based study by Baghdadi et al. (2015). In contrast, in our proposed method, the attractor-merging bifurcation produced by RRO feedback signals is realized by the external stimulus, instead of reinforcement through repeated training. Therefore, the neurofeedback method based on RRO feedback signals may facilitate the development of promising neurofeedback methods for ADHD which immediately induce the enhancement of attention in a single trial of feedback signal application.

The actual external stimulus consisting of the reference signal for intended neural activity *S*(*n*) and RRO feedback signals *Cu*(*n*) to the frontal and sensory cortices must be considered. Abnormal neural activity of dopaminergic neural networks in the frontal eye field (FEF) and visual area 4 (V4) are known to cause dysfunction in covert spatial attention and selective attention in ADHD (Mason et al., 2003) (reviewed in Noudoost and Moore, 2011). Moreover, microstimulation to the FEF and V4 can induce control of covert spatial attention and selective attention (reviewed in Moore et al., 2003). This microstimulation may be considered an effective candidate for the actual external stimulus in the RRO feedback method. However, from the viewpoint of neurofeedback, the application of stimuli using invasive methods is unsuitable. In this regard, the presentation of a blue-light stimulus to the eyes has been reported to affect neural activity in the brainstem, including the locus coeruleus and noradrenergic neural networks (González and Aston-Jones, 2006; Vandewalle et al., 2007). Moreover, the use of a blue-light stimulus reportedly enhances neural activity in right-hemisphere attention networks (Perrin et al., 2004; Vandewalle et al., 2006) and directivity of spatial attention (Newman et al., 2016). Therefore, the application of a blue-light stimulus may be a practical and effective candidate for implementing reference signals and RRO feedback signals in neurofeedback. Additionally, under the appropriate strength of RRO feedback signal, the synchronization to the reference signal can be achieved despite weak perturbation where the synchronization cannot be induced by only input of external stimulus (see Figures 6, 8). Consequently, RRO feedback signals might lead the lower invasive neurofeedback method in comparison with the case using only periodic stimulation.

To reduce abnormal CCI of neural activity, a synchronization mechanism was utilized through chaotic resonance produced by the RRO feedback method in this study. In addition to this approach, alternative methods to stabilize chaotic frontal neural activity should be discussed. Studies on non-linear feedback control theory have proposed various chaos-controlling methods, typified as the Ott–Grebogi–Yorke method (Ott et al., 1990), delayed feedback method (Pyragas, 1992; Nakajima, 1997), and *H*_∞_ control (Jiang et al., 2005) (reviewed in Schöll and Schuster, 2008). In particular, the delayed feedback method was utilized in neural systems because this method is realized by feedback terms based on previous targeted periodic *p* states (Rosenblum and Pikovsky, 2004; Nobukawa et al., 2020b). However, the stabilization cannot be realized under conditions of odd numbers of real characteristics of map functions multipliers, i.e., *F*^*p*^(*x*(*n*)) where *p* is an odd number and *F* corresponds to the map functions for neural activity observed in the experimental condition (Ushio, 1996; Nakajima, 1997). To determine whether this condition is to be avoided, an estimation of the detailed profile of map multipliers is necessary (Ushio, 1996; Nakajima, 1997); generally, estimating this profile from actual neural activity is challenging. In our proposed method utilizing chaotic resonance, the estimation of map multipliers is not required.

Several limitations of this study should be considered. First, comparison of the results of model-based studies, such as this study, with empirical studies is crucial to validate the proposed method. However, in this study, we used a simple neural system consisting of frontal and sensory cortices. To reproduce the neural activity in ADHD underpinned by complex neural bases, more precise and realistic neural network models are required for comparison and validation. For these evaluations, the use of spiking neural networks to reproduce realistic neural activity (Nobukawa et al., 2019a, 2020c) enhances the physiological validity of RRO feedback methods. Additionally, the model-based study with high physiological validity is critical to develop RRO feedback signals corresponding to actual sensory stimulus. In addition to modeling studies, developing physiological external stimuli to control neural activity for attention-related functions, such as the aforementioned blue-light (González and Aston-Jones, 2006; Vandewalle et al., 2007; Newman et al., 2016), is needed to implement signals to induce chaotic resonance. Moreover, the clarification of validate range of the measurement error and its influence to the RRO feedback method in the actual experimental environment regarding the modeling results shown in Figure 9 are relevant. In addition to measurement error, evaluation against delay in the process for producing RRO feedback signals is a crucial issue in the empirical conditions, because this delay might affect the ability of chaotic resonance. For these evaluations, the experimental studies using EEG are needed. Future research should pursue these avenues.

In conclusion, this model-based study demonstrated that chaotic resonance controlled by the RRO feedback method induced the transition of dysfunctional frontal cortical neural activity underscoring attention deficits to approximate intended healthy activity. Although several limitations exist, our proposed neurofeedback method utilizing the mechanism of chaotic resonance produced by RRO feedback signals can be practically applied as a promising treatment option for ADHD.

## Data Availability Statement

The raw data supporting the conclusions of this article will be made available by the authors, without undue reservation.

## Author Contributions

SN, NW, HN, and TT conceived the methods. SN analyzed the results, wrote the main manuscript text, and prepared all the figures. SN and HD conducted the experiments. All authors reviewed the manuscript.

## Conflict of Interest

The authors declare that the research was conducted in the absence of any commercial or financial relationships that could be construed as a potential conflict of interest.

## Publisher's Note

All claims expressed in this article are solely those of the authors and do not necessarily represent those of their affiliated organizations, or those of the publisher, the editors and the reviewers. Any product that may be evaluated in this article, or claim that may be made by its manufacturer, is not guaranteed or endorsed by the publisher.
